# Scaling bioinformatics applications on HPC

**DOI:** 10.1186/s12859-017-1902-7

**Published:** 2017-12-28

**Authors:** Mike Mikailov, Fu-Jyh Luo, Stuart Barkley, Lohit Valleru, Stephen Whitney, Zhichao Liu, Shraddha Thakkar, Weida Tong, Nicholas Petrick

**Affiliations:** 1 Office of Science and Engineering Labs, Center for Devices and Radiological Health, US Food and Drug Administration, 10903 New Hampshire Ave., Silver Spring, MD 20993 USA; 2 Division of Bioinformatics and Biostatistics, National Center for Toxicological Research, U.S. Food and Drug Administration, 3900 NCTR Road, Jefferson, AR 72079 USA

**Keywords:** HPC, Blast, Parallelization, MPI, Multi-threading, Bioinformatics, Array jobs, Next generation sequencing, Grid engine, Cluster

## Abstract

**Background:**

Recent breakthroughs in molecular biology and next generation sequencing technologies have led to the expenential growh of the sequence databases. Researchrs use BLAST for processing these sequences. However traditional software parallelization techniques (threads, message passing interface) applied in newer versios of BLAST are not adequate for processing these sequences in timely manner.

**Methods:**

A new method for array job parallelization has been developed which offers O(T) theoretical speed-up in comparison to multi-threading and MPI techniques. Here T is the number of array job tasks. (The number of CPUs that will be used to complete the job equals the product of T multiplied by the number of CPUs used by a single task.) The approach is based on segmentation of both input datasets to the BLAST process, combining partial solutions published earlier (Dhanker and Gupta, Int J Comput Sci Inf Technol_5:4818-4820, 2014), (Grant et al., Bioinformatics_18:765-766, 2002), (Mathog, Bioinformatics_19:1865-1866, 2003). It is accordingly referred to as a “dual segmentation” method. In order to implement the new method, the BLAST source code was modified to allow the researcher to pass to the program the number of records (effective number of sequences) in the original database. The team also developed methods to manage and consolidate the large number of partial results that get produced. Dual segmentation allows for massive parallelization, which lifts the scaling ceiling in exciting ways.

**Results:**

BLAST jobs that hitherto failed or slogged inefficiently to completion now finish with speeds that characteristically reduce wallclock time from 27 days on 40 CPUs to a single day using 4104 tasks, each task utilizing eight CPUs and taking less than 7 minutes to complete.

**Conclusions:**

The massive increase in the number of tasks when running an analysis job with dual segmentation reduces the size, scope and execution time of each task. Besides significant speed of completion, additional benefits include fine-grained checkpointing and increased flexibility of job submission. “Trickling in” a swarm of individual small tasks tempers competition for CPU time in the shared HPC environment, and jobs submitted during quiet periods can complete in extraordinarily short time frames. The smaller task size also allows the use of older and less powerful hardware. The CDRH workhorse cluster was commissioned in 2010, yet its eight-core CPUs with only 24GB RAM work well in 2017 for these dual segmentation jobs. Finally, these techniques are excitingly friendly to budget conscious scientific research organizations where probabilistic algorithms such as BLAST might discourage attempts at greater certainty because single runs represent a major resource drain. If a job that used to take 24 days can now be completed in less than an hour or on a space available basis (which is the case at CDRH), repeated runs for more exhaustive analyses can be usefully contemplated.

## Background

One of the most widely used bioinformatics applications is Basic Local Alignment Search Tool (BLAST) from the National Institute of Health [1]. BLAST [2] and its many variants (BLASTN, BLASTP, BLASTX, BLASTZ, etc.) are used by more scientists than any other bioinformatics application [3]. The BLAST family of programs is used to address a fundamental problem in bioinformatics research: sequence search and alignment. Using these programs scientists compare query sequences with a library or database of sequences like GenBank [4] to identify library sequences that resemble each query sequence. To be practical, developers of BLAST apply a heuristic algorithm using a statistical model to speed up the search process and achieve linear time complexity. This approach produces less accurate results than the exhaustive Needleman-Wunch [5] and Smith-Waterman [6] algorithms created earlier for the same purposes. These exhaustive algorithms are based on dynamic programming and have time complexity of *O*(*n* ^2^). They are therefore problematic for practical use in resource-constrained environments.

**Fig. 1:**
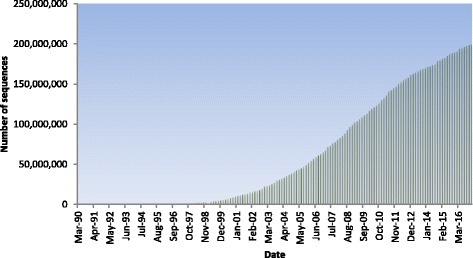
*Exponential growth of GenBank* [4]*.* The number of nucleotide sequences stored in GenBank is growing rapidly. Currently the database holds more than 200 K nucleotide sequences. The size of such reference databases poses a great challenge for the bioinformatics community to get job completed with available memory in available computational equipment

Recent developments in molecular biology and next generation sequencing technologies have led to the exponential growth of the sequence database. Figure 1 shows the exponential growth of the GenBank database, an annotated collection of all publicly available DNA sequences. Growth in the last 13 years has been thirteen fold. The BLAST family of algorithms has been slow keeping up with the current rate of sequence acquisition [7].

Parallel versions of BLAST – mpiBLAST and BLAST+ − have been developed using MPI [8] and Pthreads [9] to meet the challenges of the growing number of sequences. Nisha Dhanker et al. [1] have investigated the performance of the parallel implementations of the BLAST algorithm in HPC environments. Their research indicates that starting with release 1.6, mpiBLAST improves BLAST performance by several orders of magnitude through database fragmentation, query segmentation, intelligent scheduling and parallel I/O.

However, multi-threading using Pthreads is confined to only one node, while MPI suffers from its centralized architecture – only one master core serves potentially thousands of worker nodes causing congestion and slowing execution times. Dhanker et al. [1] address in detail the scalability limits of mpiBLAST and recommend a combination of enhancements such as a software remediation using mpiBLAST-PIO as well as hardware power in the form of 40Gb InfiniBand networking. Most intriguingly, Dhanker et al. also investigate segmentation of both the query database and the reference database, but did not fully explore its potential using the array job technique.

The other shortfalls of MPI include the lack of checkpointing to reduce the portion of a job that needs to be rerun in case of a system failure. MPI also limits scaling to the maximum number of cores available in the cluster. Finally, if the database cannot be completely cached in the available memory of a computing node, performance decreases drastically.

All BLAST applications perform pair-wise comparisons of query and database sequences. This has led to separate research into “query slicing” [10] and “database splitting” [11] to speed up performance on HPC clusters. The query slicing and database splitting approaches are exciting partial solutions. The dual segmentation approach explored on the CDRH HPC combines both approaches to take advantage of their respective benefits.

It should be noted that when using the database splitting approach [9] with some of the BLAST+ family applications (for instance, BLASTN), the application requires the effective number of sequences in the original database being split so it can compute the Expect value (E) while processing each database fragment [12]. However, BLAST+ does not provide an input parameter option for specifying the number of sequences. The database splitting approach in [11] does not offer a solution for cases where E needs to be computed. Our effort to improve BLAST HPC performance includes a modification of the BLAST+ source codes to provide this option.

## Methods

### Current BLAST application architecture and deficiencies for parallelization

**Fig. 2:**
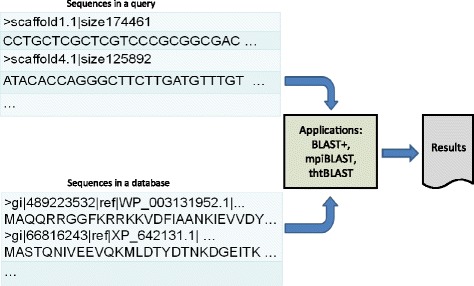
*Current application architecture.* The sequences in the query and the reference database are mapped using BLAST+, mpi BLAST and thtBLAST. These applications perform pair-wise comparisons of the sequences and conduct alignment to produce the matched output

Figure 2 shows the current application architecture for the BLAST+ family of programs along with mpiBLAST and tntBLAST [13]. In all cases, sequences in a query and a database are given to the application as input data; the application conducts alignment and search using pair-wise comparison of sequences in the query and database, and finally it outputs the matches. Table 1 summarizes the drawbacks of parallelization techniques implemented by these applications.

**Table 1:** Drawbacks of traditional parallelization techniques

Parallelization Technique	Application	Drawback
Multi-threading	BLAST+	Scaling is limited to cores on one computing node.
MPI	mpiBLAST, tntBLAST	• Scaling is limited by the master/core bottleneck - one master core serves all worker cores, which may number in the thousands. • Cannot scale beyond the available cores in the cluster.
Any of the above	Any of the above	Performance degrades drastically if the sequences in queries and database cannot completely fit in the memory of the computing node.
Any of the above	Any of the above	No checkpointing. Failures during long job runs can mean serious schedule setbacks, a risk that can negatively affect research design and dilute research objectives.

### BLAST+ enhancement to allow specification of number of database sequences

The source codes of BLAST+ can be modified as shown below to add an input option dbseqnum (“Effective number of sequences in the database”) for specifying the effective number of sequences in the original database. For this study the source codes of BLAST+ in *ncbi-blast-2.3.0 + −src.tar.gz* file were downloaded from ftp://ftp.ncbi.nlm.nih.gov/blast/executables/blast+/2.3.0/ and used for the modifications.

**Add line #60 in ncbi-blast-2.3.0 + −src/c++/src/algo/blast/blastinput/cmdline_flags.cpp file:**

const string kArgDbSeqNum(“dbseqnum”);

This modification defines a new BLAST+ command line flag/option dbseqnum.

**Add line #60 in ncbi-blast-2.3.0 + −src/c++/include/algo/blast/blastinput/cmdline_flags.hpp file:**

NCBI_BLASTINPUT_EXPORT extern const string kArgDbSeqNum;

This modification exports (makes visible) the new command line definition to other source files.

**Add lines ## 1763–1766 in ncbi-blast-2.3.0 + −src/c++/src/algo/blast/blastinput/blast_args.cpp file:**

// DB sequence number.

arg_desc.AddOptionalKey(kArgDbSeqNum, “num_sequences”,

"Effective number of sequences in the database ",

CArgDescriptions::eInteger);

This modification adds the dbseqnum optional key to command line arguments.

**Add lines ## 1938–1940 in ncbi-blast-2.3.0 + −src/c++/src/algo/blast/blastinput/blast_args.cpp file:**

if (args[kArgDbSeqNum]) {.

opts.SetDbSeqNum(args[kArgDbSeqNum].AsInteger());

}

This modification sets the dbseqnum value in the program.

After the above modifications, BLAST+ can be built following the standard build instructions in the BLAST+ documentations.

### Proposed dual segmentation method architecture

The proposed method consists of the following high-level steps:

1. Use the –info option of the blastdbcmd program (part of the BLAST+ package) to find the number of sequences (dbseqnum) in the original databases. The blastdbcmd –info option returns an integer. Record the number for use later.
2. Split the query and reference databases into M and N subsets respectively. The extent of splitting of the databases must be sufficient so that the combined size of every M and N subset pair is small enough to be cached in the memory of any computing node that will be used during the job execution.
3. Generate the unique pairs of query and database subsets.
4. Form and launch an array job of M x N pseudo-parallel tasks on your HPC cluster and provide every task with a unique pair of query and database subsets. Every task produces the partial result for its unique pair.
5. Aggregate/merge the partial results after all tasks are completed.

For a test database hs58179009.fasta the blastdbcmd program outputs the following:

“Effective number of sequences in the database is **523449** ; effective database size is **2457100615** .”

Splitting the query and database can be efficiently accomplished using the –pipe functionality of the open source GNU Parallel [14] or FASTA Splitter [15] command-line utilities. The command shown below uses GNU Parallel and splits (in parallel using eight CPUs) a test query file query.fasta into subsets of maximum size 100,000 KB each, and places them into files named query_1, …, query_152 (M = 152). The generated subsets are placed in the folder query/split:

cat query.fasta | parallel –j 8 –block 100,000 k --recstart ‘>‘--pipe tee \.

query/split/query_{#} > /dev/null.

Shown below is a serial job script file that accomplishes the query splitting task on an HPC cluster using the open source Son of Grid Engine (SGE) [16]:

#$ -cwd.

#$ -S /bin/sh.

#$ -o /dev/null.

#$ -l h_vmem = 2G.

#$ -N split_query.

#$ -pe thread 8.

time cat query.fasta | parallel -j $NSLOTS --tmpdir tmp --block 100,000 k \.

--recstart ‘>‘--pipe tee query/split/query_{#}.

Using eight CPUs in parallel, the above operation took **less than a minute** to split a 15 GB FASTA file query.fasta into 152 subsets each of size 100,000 KB maximum.

A similar GNU Parallel script was used to split the database into 27 subsets db_1, …, db_27 (*N* = 27), also of maximum 100,000 KB size each. The segments are placed in the folder db/split.

The SGE array job script that processes the M x N pairs thus produced is shown below:
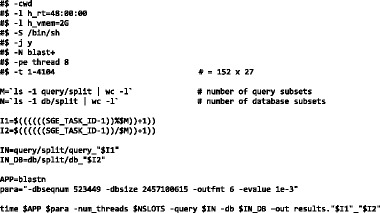


This script performs the following operations:

- Launches 4104 (152 × 27) tasks in pseudo-parallel manner.
- For every task, determines (using unique task ID, SGE_TASK_ID) a unique pair of query and database subsets and populates the $IN and $IN_DB variables respectively.
- For every task, forms additional command line options in the $para variable which also includes the dbseqnum.
- For every task, runs blastn on the unique pair of query and database subsets.

Table 2 shows how the array job script generates unique subset pairs (I1, I2) based on SGE_TASK_ID. For each of the 27 database subsets in the I2 row (I2 ranges from 1 … 27), there are 152 pairs formed with query.fasta subsets in row I1:

**Table 2:** Generating unique subset pairs (I1, I2) based on SGE_TASK_ID

	task ID								
SGE_TASK_ID	1	2	3	…	152	153	154	…	4104
I1	1	2	3	…	152	1	2	…	152
I2	1	1	1	…	1	2	2	…	27

In the array job used for illustration, the slowest task took **less than seven minutes** to complete. All tasks ran in multi-threaded mode using eight CPUs per task. **This entire array job** could be run in less than seven minutes with the availability of 32,832 (=4104 × 8) CPUs.

**Fig. 3:**
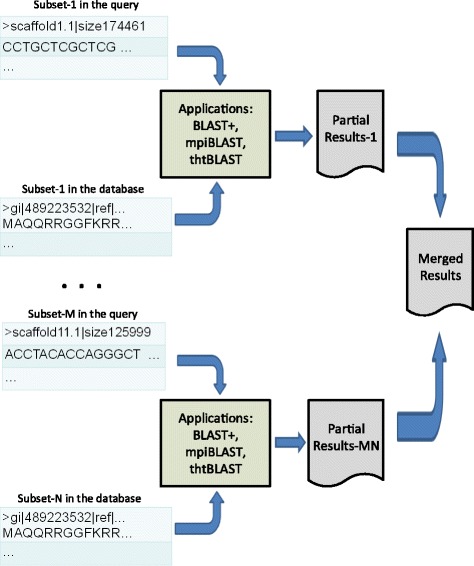
*Scalable application architecture.* For effective use of time and space, this manuscript proposes a scalable method. Where query and reference databases are split into M and N subsets respectively. Unique pairs of query and database subsets were generated. An array job of M x N pseudo-parallel tasks takes place on the HPC cluster produces partial result for its unique pair by every task. After completing all the tasks, all the generated partial results were aggregated or merged

Figure 3 demonstrates the scalable application architecture.

### Result analysis and checkpointing

The SGE array job mechanism provides natural checkpointing: if any tasks fail then only those tasks need to be rerun to recover failed partial results. Following job run completion, all the M x N partial result files for the array job tasks must be checked for having been produced. In the above example the result files are named uniformly based on the unique pair as results.“$I1”_“$I2”. The following steps reveal if any partial result files were not generated:

1. Create a list of all partial result files generated by the job run. For instance, the Linux command line below generates a list of all partial result file names located in the current working directory and places the list in the file res.txt.

ls -1 results.* > res.txt.

2. Create a list of expected partial result file names. The list can be automatically generated either before or after running the array job script. To generate this list, change to a different directory and run the array job script with the last line in the script replaced as follows:

touch results.“$I1”_“$I2”.

With this change, the job simply creates a set of empty files with the same names as the expected partial result files when the array job is executed.

To collect all the expected partial result file names in a single file named expect.txt, run the below command within this other directory:

ls −1 results.* > expect.txt.

3. Finally use the command below to compare the lists of expected and actual results files, and identify missing files:

sdiff expect.txt res.txt| grep ‘[<]’ | awk -F: ‘{print “changed: ”$1}’

The output from this command look like the sample lines below and will be in standard Linux “diff” file format:

changed: results.1_2 <.

changed: results.151_1.

4. The two lines in the above example indicate that two partial result files are missing: for unique pairs (1, 2) and (151, 1). The unique task IDs corresponding to these pairs are 153 and 151 respectively and found using the below formula:

SGE_TASK_ID = (I2 - 1) * M + I1.

5. Create a text file (name it, for instance, failed.lst) and place the failed unique task IDs in this file (one task ID per line).

Steps (3), (4) and (5) can be automated using, for instance, a Linux shell script.

The array job must then be re-run for only those missing pairs. Modify the above SGE script as shown below and rerun it.
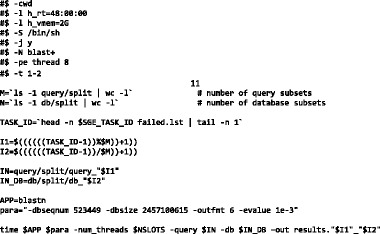


In the current example, the SGE script runs an array job of only two tasks corresponding to the two failed partial results.

### Search against *nt* database

A query test file of 15 GB was used to search against the NCBI *nt* database [17] of 126 GB using the proposed technique. The query file and *nt* database were split into 152 and 136 segments respectively. Characteristics of the query test file and *nt* database are shown in Table 3.

**Table 3:** Characteristics of the query test file and *nt* database

	*nt* database	Query test file
Number of sequences	39,204,206	73,102,023
Number of total bases	128,339,311,604	10,209,633,848
Longest sequence, base	774,434,471	151
Size, GB	126	15
Number of segments	136	152
Segment size, MB	1000	96

**Fig. 4:**
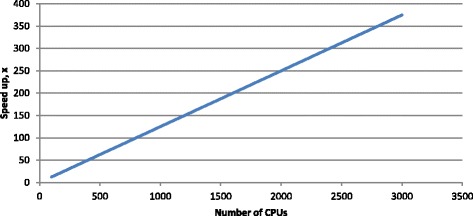
*CPU performance.* CPU shows the linear speed up as the number of available CPUs or number of tasks running in parallel increases

An array job of 20,672 (=152 * 136) tasks was created and launched as described earlier. The slowest task took less than 30 min (28 min 51.105 s) to complete. Average completion time per task was 3 min 53 s. Every task was assigned eight CPUs. On the HPC with a limited number of CPUs available at a time (3000), all tasks completed in less than 4 hours (3 h 33 min 41 s). Without applying the proposed technique the whole job would take more than 55 days (1335 h 31 min 16 s which is equal to the sum of the completion times of all 20,672 array job tasks) using eight CPUs. Figure 4 shows the linear speed up as the number of available CPUs (or number of tasks running in parallel) increases.

## Results and discussion

Searching for genome sequence similarities is one of the most important bioinformatics tasks. Bioinformatics applications such as BLAST+, DIAMOND [18], VSEARCH [19] and USERACH [20] find matches via pair-wise comparisons of sequences in queries and reference databases. The number of sequences that need to be compared is a major challenge requiring process parallelization on HPC clusters. Multithreading and/or MPI are typical parallelization techniques in use. However, these approaches are often inadequate: (a) multi-threading is confined to a single node, and (b) the single master in MPI’s centralized architecture limits scalability. An alternative O(T) architecture, where T = M x N, developed by CDRH offers significant processing speed-up and scalability for the analyses, and provides checkpointing benefits. The approach consists of processing sequence analyses in array mode with segmented query and database sets, which can dramatically reduce job runs from months to hours. The inherent checkpointing feature of the dual segmentation mode is a benefit that can hardly be overstated. The checkpointing is free, a part of the method itself.

These techniques could be applied to the exhaustive Needleman-Wunch [5] and Smith-Waterman [6] algorithms to produce even more accurate results than with the BLAST+ family of algorithms.

The increased flexibility of job submission may be useful in commercial cloud services, allowing the use of spot queues (also known as “preemptive” queues) which are typically offered at extremely low prices.

## Conclusions

The techniques presented in this study are already in use by Food and Drug Administration scientists. The segmentation of both the query database and the reference database, rather than just one or the other, enables reduction of the data subset processed by each job task to a size that fits into the memory of the computing nodes where computations are performed. The resulting reduction in disk I/O produces excellent, even stunning, results, enabling drops in BLAST run times from periods such as 27 days to a single day or even a few hours. The described method uses only open source code and adds no hardware cost.

Further experimental and theoretical studies are needed to increase automation of the techniques and broaden their applicability to more bioinformatics applications.
